# Present and future perspectives on immunotherapy for advanced renal cell carcinoma: Going to the core or beating around the bush?

**DOI:** 10.15586/jkcvhl.2015.24

**Published:** 2015-04-04

**Authors:** Hidenori Kawashima, Yasunori Kimura

**Affiliations:** Shirahama Hamayu Hospital, Shirahama, Wakayama 649-2211, Japan

## Abstract

Metastatic lesions of renal cell carcinoma (RCC) occasionally regress spontaneously after surgical removal of the primary tumor. Although this is an exceptionally rare occurrence, RCC has thus been postulated to be immunogenic. Immunotherapies, including cytokine therapy, peptide-based vaccines, and immune checkpoint inhibitors have therefore been used to treat patients with advanced, metastatic RCC. We review the history, trends, and recent progress in immunotherapy for advanced RCC and discuss future perspectives, with consideration of our experimental work on galectin 9 and PINCH as promising specific immunotherapy targets.

## Introduction

Metastatic lesions of renal cell carcinoma (RCC) occasionally regress spontaneously following removal of the primary disease, though this is exceptionally rare. This is thought to represent an immune response, and RCC, like melanoma, has thus been considered to be immunogenic. Although very few urologists have been fortunate enough to experience such a rare event, these anecdotes suggest that RCC may be immunogenic in nature. Before the era of molecular targeting drugs, cytokine therapy with interferon (IFN) or interleukin-2 (IL-2) was widely used, and partial response (PR) and complete response (CR) were achieved in 15.9% and 1.8%, respectively, of patients with advanced RCC treated with IFN (1). Despite the low response rate, it is noteworthy that cytokine therapy can achieve a CR or cure in patients with advanced metastatic RCC.

Furthermore, even though prostate cancer, unlike RCC, is not generally described as immunogenic, we experienced a patient with castration-resistant prostate cancer (CRPC) with bone metastases who maintained CR for a long period after hormonal manipulation and chemotherapy with docetaxel. In another case of CRPC, the tumor was reported to vanish spontaneously during therapy (2). These outcomes could be attributed to an immune response. These rare but important clinical observations suggest that specific cancer immunity can be triggered by certain conditions, and that cancer immunity has the potential to achieve a cure in some cases. Immunotherapy has thus been a focus of research for advanced RCC, even in the era of molecular targeting drugs.

## History of therapies for RCC

The history of therapies for RCC is summarized in **Table 1** (3). IFNα and IL-2 were approved in Japan in 1987 and 1999, respectively, and reduced-intensity stem cell transplantation (RIST) was reported by Child et al. (4) in 2000. This ‘mini-transplantation’, aimed to damage tumor cells via a graft versus host reaction, was expected to demonstrate good clinical efficacy; however, the results in Japan have been disappointing (5).

**Table 1.** History of therapy for renal cell carcinoma (Revised from 3)

Year	Notable Events
1963	Radical nephrectomy
1987	IFN-α approved (in Japan)
1990~	Partial nephrectomy for small RCC
1993	Identification of von Hippel-Lindau tumor suppressor gene: rationale for molecular targeting drugs
1999	IL-2 approved (in Japan)
2000	‘Mini-transplantation’ (reduced-intensity stem cell transplantation; RIST) for treatment of RCC
2002	Laparoscopic nephrectomy approved (in Japan)
2008	Molecular targeting drugs approved (in Japan)

Identification of the von Hippel-Lindau (VHL) tumor suppressor gene in 1993 (6) and subsequent discovery of a high rate of VHL gene mutations in patients with sporadic RCC (7) formed the theoretical basis for molecular targeting drugs for advanced RCC, such as tyrosine kinase and mammalian target of rapamycin inhibitors. The VHL gene product functions as an ubiquitin ligase targeting hypoxia-inducible factor-1α (HIF-1α). HIF-1α levels are regulated by its degradation in the ubiquitin/proteasome system. Excess HIF-1α caused by malfunctioning of the VHL protein results in up-regulation of vascular endothelial growth factor (VEGF) and glucose transporter-1, which are responsible for the characteristic RCC phenotypes of hypervascularity and clear cytoplasm on hematoxylin and eosin staining, respectively.

Molecular targeting drugs target the cascade mediated by VHL protein, and several such agents, including sorafenib, sunitinib, axitinib, temsirolimus, and everolimus, have been approved in Japan since 2008.

## Therapeutic approaches for RCC: immunotherapy and molecular targeting drugs

The extremely rare spontaneous regression of metastatic lesions following surgical removal of the primary lesion, e.g., by nephrectomy, is thought to be caused by an immunologic response. This characteristic of RCC and the consequent notion that RCC is immunogenic, form the basis for non-specific immunotherapy with cytokines, which has been employed for some time. However, molecular targeting drugs currently form the mainstream of treatment for metastatic RCC. The use of IFNα is currently limited to lung metastases. In this review, we consider recent progress in immunotherapy for RCC and discuss future prospects with regard to the role of immunotherapy as a mainstream therapy for RCC.

## Overview of cytokine therapy for advanced, metastatic RCC

Urologists have continued to treat patients with advanced metastatic RCC using IFNα for the past 20 years. Although a previous study reported response rates to IFNα as low as 15.9% and 1.8% for PR and CR, respectively (1), it is noteworthy that cytokine therapy resulted in some instances of CR, which were not achieved with molecular targeting drugs. Furthermore, a Japanese multicenter study reported a survival benefit of cytokine therapies in RCC patients with lung metastases (8). Cytokine therapy may thus be generally beneficial in many patients with RCC. The combination of IFN and IL-2 resulted in response rates of 35.7% and 4.8% for CR+PR and CR, respectively, indicating further possibilities of cytokine therapy (9).

The anti-tumor mechanisms of IFN involve activation of macrophages and monocytes, enhancement of natural killer (NK) cell activities, induction of antigen presentation on the cell surface, and enhancement of cytotoxic T lymphocyte (CTL) activities (10). Although IL-2 enhances the activities of NK cells, B cells and T cells, including CTLs, it also temporarily activates regulatory T cells (Tregs) via the IL-2 receptor α-chain on Tregs, and may thus suppress CTL activities with possible unfavorable effects.

## Peptide-based vaccines

Peptide-based vaccines represent a rational approach to inducing cancer-specific immunity against cancer antigens. Tumor-associated antigens (TAA) are incorporated into antigen-presenting cells (APCs), broken down into pieces, processed, and presented on HLA class I (MHC class I) in the form of peptides. The presented peptide antigens stimulate CD8+ effector cells (CTLs) that specifically recognize the antigen and attack cancer cells bearing the TAA. The amino acid sequences of the peptides within TAA sequences have been investigated with the aim of producing peptide-based vaccines. Candidate peptides capable of binding HLA class I are synthesized and used to stimulate peripheral blood mononuclear cells in vitro to induce antigen-specific CTLs. Peptides capable of inducing CTLs with high, specific cytotoxic activity toward cancer cells are then selected for use as vaccines. These peptides, mixed with adjuvant, are presented on HLA class I of APCs following injection, resulting in lymphocyte stimulation and induction of tumor antigen-specific CTLs.

Regarding the clinical efficacy of peptide-based vaccines for RCC, Uemura et al. (11) reported three PR and six stable diseases (SD) among 23 patients with metastatic disease in a phase I trial of carbonic anhydrase 9-derived peptides. Limited efficacy (SD) was achieved following immunotherapy using Wilms’ tumor 1 peptide in two of three RCC patients enrolled in one study (12). Hypoxia-inducible factor prolyl hydroxylase 3 (HIFPH3) was shown to be over-expressed in primary RCC tissues and many RCC cell lines, and an HIFPH3-derived peptide induced CTLs in three of six RCC patients, though no clinical trials have been reported (13).

A phase II randomized, clinical trial of IMA901, a mixture of multiple TAA-derived peptides, showed that Tregs were reduced by a single dose of cyclophosphamide, and patients responded immunologically to IMA901 had longer overall survival (14). A phase I clinical trial of human VEGF receptor 1-derived peptide vaccines in patients with metastatic RCC demonstrated PR in two and SD for more than 5 months in eight of 18 patients (15).

Peptide-based vaccine therapy emulates naturally occurring antigen presentation, and targets specific cellular immunity against cancer cells using TAA-derived peptides. Peptide vaccine therapy is thus rational with the potential for high efficacy. However, it has so far demonstrated limited clinical effects in patients with metastatic RCC, probably because of the lack of defined, appropriate antigens.

## Innate and acquired immune systems in cancer immunotherapy

The innate immune system seems to play an important role in the initial stages of anti-cancer immunity. Concerning the implications of innate immunity for immunotherapy of RCC, several reports have focused on NK cells. Combination therapy with IFNα and IL-2 has been reported to enhance the cytotoxic activities of NK cells in patients with advanced RCC (16) and NK cells have been shown to be necessary for anti-tumor activities in RCC patients treated with cytokines (17). Furthermore, low numbers of peripheral NK cells (NK-Kir+) correlated with shorter disease-free survival in patients with RCC (18).

NKT cells also contribute to anti-cancer immunity, and a lack of these cells is associated with impaired anti-cancer immunity (19). The exogenous NKT cell ligand α-galactosylceramide was tested in a clinical trial for lung cancer and head and neck cancer (20). NKT cells are activated as follows: Toll-like receptors activate dendritic cells to produce IL-2. Stimulation by IL-2 and recognition of a self-ligand presented on CD1d of APCs then cause activation of NKT cells, which in turn activate specific helper T cells, together with CD8+ CTLs. The NKT cell system thus acts as a functional bridge between the innate and acquired immune systems in the development of specific immunity. The innate immune system, represented by the NK and NKT systems, thus provides an initial step in anti-cancer immunity eventually leading to acquired and cancer-specific immunities.

It is likely that the development of specific and effective immunity against RCC could achieve a cure, though no effective means of achieving this goal have yet been identified.

## Galectin 9 and PINCH: isolation and evaluation of new tumor antigens for peptide-based vaccine therapies

Despite a low response rate, cytokine therapy can offer an effective therapeutic option for advanced RCC. Survival benefit of cytokine therapy has been reported in patients with metastatic RCC (8), and the observation that cytokine therapy can induce a CR in a very limited number of patients with metastatic RCC indicates the curative potential of immunotherapy. The key to understanding the successful induction of anti-cancer immunity involves knowing which antigens are important and how specific immunity is acquired in those few patients who respond well to cytokine therapy. It is also important to establish if the mechanisms of specific immunity in those successful cases are applicable to RCC patients in general. If so, general methods for inducing cancer immunity based on these specific cases may provide effective and potentially curative therapies for patients with advanced RCC. This could reduce the need for expensive, molecular targeting drugs, thus helping to limit the expanding medical expenditure in Japan.

**Figure 1.**
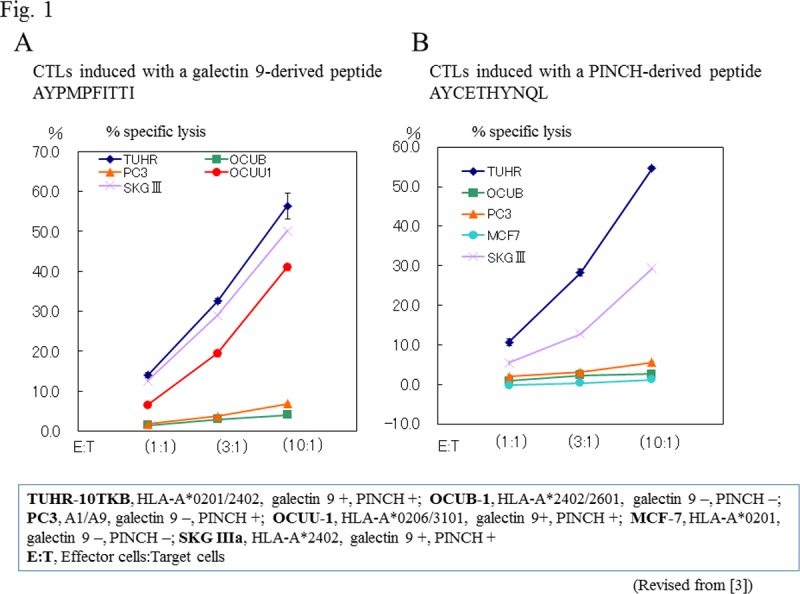
Antigen-specific, HLA-A*2402-restricted cytotoxicities of CTLs induced with galectin 9-derived (A) and PINCH-derived peptides (B). A, B. Peripheral blood mononuclear cells from healthy volunteers (HLA-A*2402) were stimulated with galectin 9-derived and PINCH-derived peptides, respectively, to induce CTLs. The cytotoxicity of the CTLs toward RCC cells was assessed by a 51Cr-release assay (21).CTLs induced by both peptides showed high cytotoxic activity toward TUHR-10TKB RCC cells (HLA-A*2402+, galectin 9+, PINCH+) and SKGIIIa uterine cancer cells (HLA-A*2402+, galectin 9+, PINCH+). CTLs were not active toward cells with different HLA-A or negative antigens. Bars represent means±standard errors of assays performed in triplicate. A. CTLs induced by the galectin 9-derived peptide exhibited cytotoxicity toward OCUU-1 RCC cells (HLA-A*0206+, galectin 9+), implying less stringency in terms of HLA-A for the CTLs used.

We investigated ‘true cancer antigens’ by screening an RCC expression library using sera from patients with metastatic disease who responded well to cytokine therapy as probes, under the assumption that these responders had acquired specific antibodies against RCC together with specific cellular immunity. We identified two novel genes, galectin 9 and PINCH, as RCC-specific antigens that were specifically highly expressed in all the tested clear cell carcinoma samples, compared with normal renal tissues (21). Using peptides derived from these two antigens, we induced HLA-A*2402-restricted CTL clones and HLA-A*0201-restricted CTLs with high antigen-specific killing activities toward RCC cells (21) **(Figure 1)**. These antigens seemed to be closely related to an immune escape mechanism, cancer cell survival, and metastases of RCC.

Galectin 9 modulates cellular immunity by suppressing excess immune reactions such as allergies. Its receptor is T cell immunoglobulin and mucin domain 3 (TIM-3), located on the surface of T cells. Galectin 9 causes apoptosis of activated T cells through TIM-3 (22, 23), which is an immune checkpoint molecule, as are programmed cell death protein-1 (PD-1) and CTL-associated protein 4 (CTLA-4) (24). These immune checkpoint molecules mediate immunosuppressive signals causing inactivation of T cells. Anti-cancer drugs targeting these immune checkpoints are currently under development, and have attracted considerable attention and expectations (25).

PINCH contributes to apoptosis resistance in cancer cells (26) and promotes epithelial-mesenchymal transition in renal tubular cells (27), and thus plays an important role in cancer survival and metastases.

Galectin 9 and PINCH produce favorable environments for RCC cells and therefore provide promising targets for immunotherapy. Our method of identifying potentially useful cancer antigens was unique in that we screened for antigens with central roles in developing specific anti-cancer immunity by reacting them with sera from responders to cytokine therapy. The abilities of the identified antigens to induce CTLs with specific, high cytotoxicity was then evaluated. This method involved basic molecular cloning techniques using clinical samples, and was developed by a urologist with extensive experience, including many clinical cases of RCC (21).

Peptide-based vaccines have the problem that various peptide sequences of the same antigen need to be prepared in accordance with the HLA types of the patients. TAAs are presented on HLA class I in a form of peptides that comprise part of the whole antigen: different peptides derived from the same whole antigen associate with HLA class I of different HLA types. The binding motif of the antigen for each HLA type should be predicted in silico, and the ability of each predicted peptide to induce CTLs by stimulating lymphocytes of the same HLA type needs to be tested. We previously identified galectin 9- and PINCH-derived peptides that induced HLA-A*02-restricted CTLs, HLA-A*24-restricted CTLs, and HLA-A*33-restricted CTLs (28), respectively, all of which exhibited specific and highly cytotoxic activities toward RCC cells. The frequency of HLA-A*02 is high in Europe and North America, while HLA-A*24 is a major HLA type, and HLA-A*33 is also common in Japan. These peptides thus represent important candidates for future peptide-based vaccine therapies.

## New anti-cancer drugs targeting immune checkpoint molecules

Cancer cells have recently been shown to exploit immune checkpoint molecules that mediate the suppression of immune signals, thus facilitating escape from the immune surveillance system. These checkpoint molecules, including CTLA-4, PD-1, programmed death ligand 1 (PD-L1), and TIM-3, mediate T cell suppression. Drugs (antibodies) targeting these check point molecules, referred to as check point inhibitors, including antibodies against CTLA-4, PD-1, and PD-L1, have been developed (25) and in some cases approved **(Table 2)**.

**Table 2.** Anti-cancer drugs targeting immune regulatory checkpoints (Revised from 3)

Drug	Target	Cancer Type	Phase of clinical trial
Ipilimumab	CTLA-4 (antibody)	Melanoma	Approved
Tremelimumab	CTLA-4 (antibody)	Hepatocellular carcinoma (hepatitis C)	II
Nivolumab (BMS-936558) MDX-1106 ONO-4538	PD-1 (antibody)	RCC, melanoma, NSCLC*	III
MEDI-4736 (Zeneca)	PD-L1 (MAb)	Solid tumors	II (lung)
MEDI-6469 (Zeneca)	OX-40 (activation: agonistic antibody)	Solid tumors	I
MPDL-3280A (Genentech)	PD-L1 (Mab, humanized)	NSCLC, melanoma, RCC	II (NSCLC)

* NSCLC, non-small cell lung cancer.

Clinical studies of an anti-PD-1 antibody (nivolumab; BMS-936558) (29, 30) and a humanized monoclonal antibody against PD-L1 (MPDL-3280A) (30) have been reported in patients with RCC. A response rate (PR + CR) of 27% was achieved with nivolumab in patients with RCC (29).

Increasing attention has been paid to TIM-3 as a target (24). TIM-3 binds to its ligand galectin 9, causing suppression of activated T cells (22, 23). Combined therapy with anti-TIM-3, anti-PD-1, and anti-CTLA-4 antibodies demonstrated an additive anti-tumor effect in experimental animal models (31). Our study suggested that galectin 9-derived peptides would induce CTLs that attack RCC, and also remove the immunosuppressive environment caused by activation of the immune checkpoint TIM-3.

## Summary

Progress in molecular biology following the discovery of the VHL gene has resulted in the development of various molecular targeting drugs and advancements in therapies for advanced RCC. On the other hand, following on from the long-standing use of cytokines such as IFN and IL-2, immunotherapy is about to enter a new era represented by immune checkpoint inhibitors. However, the costs associated with the clinical study, development, and approval of these immune checkpoint inhibitors, marketed as humanized antibodies, are likely to be enormous.

Peptide-based vaccine therapy aims to induce specific immunity, and appears to offer potential survival benefits and cure; however, their efficacy is currently poor. Innate immunity provides an initial step leading to specific anti-cancer immunity, and non-specific immune reactions initiated by cytokines may also cause specific anti-tumor immunity. The combination of ‘real’ cancer antigens and new, immune-activating agents targeting molecules such as immune checkpoints may thus induce strong cancer-specific immunity.

Cytokine therapy is highly effective in a very limited number of patients, indicating the high potential of immunotherapy. However, the mechanisms responsible for the development of immunity in these patients with RCC remain unclear. We identified novel RCC antigens that reacted with sera from cytokine-therapy responders. We successfully induced antigen-specific, HLA-restricted CTLs with high activities by stimulating lymphocytes with peptides derived from those antigens. However, the mechanisms responsible for the effects of the cytokines in particular cases are unknown. Further, detailed investigations of successfully treated cases and analysis of the general mechanisms are likely to lead to promising new therapies for RCC.
